# Public trust in the Government to control the spread of COVID-19 in England after the first wave—a longitudinal analysis

**DOI:** 10.1093/eurpub/ckad148

**Published:** 2023-08-14

**Authors:** Claudia Boehm, Paul Boadu, Josephine Exley, Mustafa Al-Haboubi, Nicholas Mays

**Affiliations:** Department of Health Services Research and Policy, London School of Hygiene and Tropical Medicine, Policy Innovation and Evaluation Research Unit (PIRU), London, UK; Department of Health Services Research and Policy, London School of Hygiene and Tropical Medicine, Policy Innovation and Evaluation Research Unit (PIRU), London, UK; Department of Health Services Research and Policy, London School of Hygiene and Tropical Medicine, Policy Innovation and Evaluation Research Unit (PIRU), London, UK; Department of Health Services Research and Policy, London School of Hygiene and Tropical Medicine, Policy Innovation and Evaluation Research Unit (PIRU), London, UK; Department of Health Services Research and Policy, London School of Hygiene and Tropical Medicine, Policy Innovation and Evaluation Research Unit (PIRU), London, UK

## Abstract

**Background:**

To control the spread of coronavirus disease 2019 (COVID-19), governments are increasingly relying on the public to voluntarily manage risk. Effectiveness is likely to rely in part on how much the public trusts the Government’s response. We examined the English public’s trust in the Conservative Government to control the spread of COVID-19 after the initial ‘crisis’ period.

**Methods:**

We analyzed eight rounds of a longitudinal survey of 1899 smartphone users aged 18–79 in England between October 2020 and December 2021. We fitted a random-effects logit model to identify personal characteristics and opinions associated with trust in the Conservative Government to control the spread of COVID-19.

**Results:**

Trust was lowest in January 2021 (28%) and highest in March 2021 (44%). Being older, having lower educational attainment and aligning with the Conservative Party were predictors of higher levels of trust. Conversely, being less deprived, reporting that Government communications were not clear and considering that the measures taken by the Government went too far or not far enough were predictors of being less likely to report a great deal or a fair amount of trust in the Government to control the pandemic.

**Conclusion:**

Trust in the Government’s response was found to be low throughout the study. Our findings suggest that there may be scope to avoid losing trust by aligning Government actions more closely with scientific advice and public opinion, and through clearer public health messaging. However, it remains unclear whether and how higher trust in the Government’s response would increase compliance with Government advice.

## Introduction

Since emerging in December 2019, the spread of SARS-CoV-2, resulting in the coronavirus disease 2019 (COVID-19) pandemic, has posed enormous challenges to public health specialists and governments around the globe. To slow the rate of transmission and avoid overwhelming health systems, many governments asked citizens to take unprecedented actions to control the spread of SARS-CoV-2 ranging from stay-at-home orders to mandatory mask wearing and vaccination.

The effectiveness of interventions largely relied on citizens to cooperate and follow public health guidance, even when mandatory since in most countries state capacity and willingness to put the population under surveillance is limited. Evidence from previous infectious disease outbreaks (SARS, H1N1, Ebola) suggests that individuals with higher trust in Government and/or Government institutions in general are more likely to adhere to public health measures.^1^^,^^2^ Early evidence on the relationship between trust in Government and compliance during the COVID-19 pandemic has demonstrated similar findings.^3–5^

Following the initial COVID-19 wave in spring 2020, many national governments sought to gradually ease restrictions. This marked a shift from regulations to guidance, with the public increasingly being asked to take greater personal responsibility to manage risk. There is considerable uncertainty about the direction the global pandemic will take. As such, governments need to be ready to respond to any resurgences and new variants, which may require the tightening of public health measures. In general, they will be relying on individuals to voluntarily follow advice to reduce their and other community members’ risks.

In crises, levels of trust in Government often follow a typical pattern: initially increasing (‘crisis effect’ or ‘rally-round-the-flag effect’), and then declining as the consequences of the crisis become clearer.^3^^,^^6^ The same phenomenon was observed during the first wave of the COVID-19 pandemic.^3^^,^^7^^,^^8^ In the context of shifting to increasing reliance on voluntary action as countries move beyond the initial crisis phase, efforts to sustain adherence to public health measures are likely to become increasingly reliant on the public’s trust in Government and its institutions.

Using eight rounds of longitudinal survey data, this study examined changes in the English general public’s trust in the Government specifically to control the spread of COVID-19 beyond the initial ‘crisis’ period. It sought to identify individual characteristics and opinions associated with higher levels of trust and to describe how changes in case numbers and Government policies affected the public’s level of trust.

## Methods

### Study design

We undertook a panel study with eight rounds of survey data collected between October 2020 and December 2021, as part of a wider study of public attitudes towards, and use of, the National Health Service (NHS) COVID-19 contact tracing smart phone application. Ethics approval for this study was obtained from the London School of Hygiene & Tropical Medicine (Reference Numbers 22483 and 25819).

### Setting

**Figure 1:**
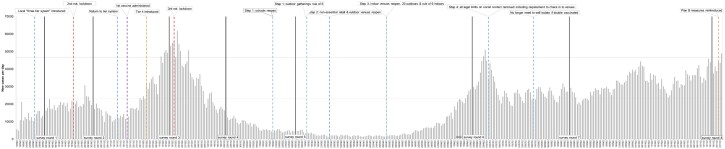
Overview of number of daily new cases and policy measures implemented in England over the course of the study. Round 1: 14 to 22 October 2020, took place after the introduction of the local three-tier system. Round 2: 12 to 23 November 2020, conducted during the second national lockdown, which was introduced on 5 November. Round 3: 28 December 2020 to 6 January 2021, conducted over the Christmas period when the country had returned to the tier system. The south east of England moved to newly created tier 4 (‘stay at home’). The first vaccine was administered in the UK on 8 December 2020. Round 4: 1 to 15 February 2021, took place during the third national lockdown, which had started on 6 January 2021. Round 5: 15 to 31 March 2021, took place at the start of the easing restrictions. The Government outlined a four-step plan, at the time of the survey schools had reopened. Round 6: 1 to 18 July 2021, took place ahead of the final step of the easing of restrictions; indoor venues had reopened, people were allowed to meet in groups of up to 30 outdoors and 6 indoors. Round 7: 31 August to 13 September 2021, took place after all legal limits on social contact had been lifted. Those who had been double vaccinated were also no longer required to self-isolate if they had come into contact with someone who tested positive for COVID-19, if they didn’t have any symptoms. Round 8: 25 November to 13 December 2021, took place at the same time as the Omicron variant was first being reported; six Southern African countries were placed on the travel red list and the first cases were detected in the UK. On 8 December the Government announced that Plan B were to be reintroduced; face coverings mandatory in most indoor settings, work from home, use of the NHS COVID pass for entry into nightclubs and settings where large crowds gather. Number of daily new cases obtained from the website: https://coronavirus.data.gov.uk/details/cases

Figure 1 provides an overview of the Government’s response to COVID-19 in England over the eight survey rounds. Most lockdown restrictions had been lifted by July 2020. However, by autumn 2020, restrictions once again began to tighten: on 14 September, people were legally prohibited from meeting more than six people socially, and on 14 October, the Government introduced a new three-tier system of restrictions.^9^ On 5 November 2020, England entered the second national lockdown, returning to the tier system at the beginning of December. On 19 December 2020, a fourth tier was introduced, before a third national lockdown was introduced on 6 January 2021 in response to the rapid spread of the Alpha variant. From March 2021, as vaccine rollout progressed, restrictions were lifted based on a four-step ‘roadmap out of lockdown’.^10^ Step four of the roadmap was delayed by 4 weeks to allow more people to receive their first dose of the vaccine. By July 2021, most legal limits on social contacts were lifted and all sectors of the economy were reopened; England opened earlier than many comparable countries. The ‘Autumn and Winter’ plan for COVID-19 was released in September 2021, setting out a Plan A and Plan B for managing the virus.^11^ In response to the Omicron variant, England moved to Plan B on 8 December 2021, which included mandatory face masks in most indoor public spaces and a legal requirement to check in to some venues.

### Participants

A representative sample of English smartphone users aged 18–79 was recruited from YouGov’s volunteer online panel, with quotas set on age, gender, region and socioeconomic status. All respondents were invited to participate in each subsequent round of the survey. Participation was entirely voluntary, and participants’ consent was obtained before completing the baseline survey.

### Data collection

The online surveys were conducted roughly every 6–8 weeks. The survey questionnaire consisted of questions developed by the research team and adapted from previous COVID-19 tracker surveys.^12^^,^^13^ The survey was primarily designed to capture participants’ views on and use of NHS England’s COVID-19 app. It additionally included a module on participants’ perceptions of the Government’s response to, and handling of, the COVID-19 pandemic. Demographic information at baseline was provided by YouGov.

### Statistical methods

The main analysis was restricted to participants who responded to all eight rounds of the survey because the balanced dataset gave a higher log likelihood coefficient than the unbalanced dataset. Our outcome of interest was the level of trust—based on the survey question ‘To what extent, if at all, do you trust the Government to control the spread of COVID-19?’ (options ‘a great deal’, ‘a fair amount’, ‘not very much’, ‘not at all’ and ‘don’t know’). Explanatory variables were selected to capture participants’ sociodemographic characteristics, and attitudes towards the pandemic and Government measures (see Supplementary table S1 for variables included in the model and their definitions).

The descriptive analysis used weighting within survey rounds (see Supplementary figure S1) so that the achieved sample was representative of the population of smartphone users in England using data from the 2019 Ofcom Technology Tracker Survey.^14^

We fitted a random-effects logit model to examine factors associated with *trust* (see Model specification in Supplementary file). The outcome of interest, *trust*, was binary, measured as 1 if a respondent reported ‘a great deal’ or ‘a fair amount’ of trust in the Government to control the spread of COVID-19 or 0 if a respondent reported ‘not very much’ or ‘not at all’. Our categorization of the dependent variable was based on the assumption that at a given point during the pandemic, respondents either had trust or no trust in the Government to control the spread of COVID-19. To ensure that there was no loss of information results from our categorization of the dependent variable, we also investigated the data using random-effects ordered logistic regression; however, the results were similar (see Supplementary tables S5 and S6). Respondents who reported ‘don’t know’ (*n* = 369 observations) and were missing data on education, index of multiple deprivation or political party affiliation (*n* = 36 individuals) were excluded from the multivariate model. All variables associated with *trust* in the univariate analysis were included in the model (see Supplementary table S3). We estimated the multivariate model recursively, dropping each variable in turns to assess the performance of the model using the Likelihood-Ratio test, to arrive at the final model (see Supplementary table S4).

## Results

In total, 1899 smartphone users aged 18–79 in England completed the baseline survey. Of these, 873 (46%) completed all eight survey rounds (see Supplementary table S2). Baseline characteristics are presented in table 1. Compared with the general population, there was overrepresentation of people with higher education (57% vs. 44%) and younger age groups in the sample.^15^^,^^16^ Since younger and more highly educated people are more likely to vote for parties other than the Conservative Party, Conservative voters are likely to be underrepresented in the sample.^17^

**Table 1:** Baseline distribution of respondent’s characteristics in the sample (survey round 1), *N* = 873

	*n*	Unweighted %	Weighted %
Gender			
Male	414	47.4	49.7
Female	459	52.6	50.3
Age category			
18–29	116	13.3	12.9
30–49	316	36.2	39.9
50–64	280	32.1	30.3
>65	161	18.4	16.9
Ethnicity			
White	776	88.9	87.8
All other ethnic groups	97	11.1	12.2
Region			
North East	46	5.3	4.4
North West	121	13.9	13.7
Yorkshire and the Humber	87	10.0	9.6
East Midlands	79	9.1	8.1
West Midlands	89	10.2	11.1
East of England	101	11.6	12.0
London	104	11.9	13.1
South East	162	18.6	19.1
South West	84	9.6	8.9
Urbanity			
Urban	696	79.7	81.0
Town and fringe	80	9.2	8.5
Rural	96	11.0	10.3
Not answered	1	0.1	0.2
Highest level of education attainment			
Lower education	225	25.8	25.3
A-level or equivalent	130	14.9	15.3
Higher education	497	56.9	56.8
Not answered	21	2.4	2.6
Employment status			
Currently working	463	53.0	54.4
Not currently working	33	3.8	4.1
Unpaid/Voluntary work	2	0.2	0.2
Look after home or family	47	5.4	5.4
Unemployed	45	5.2	5.2
Permanently sick or disabled	56	6.4	6.2
Education	20	2.3	2.8
Retired	194	22.2	20.3
Other	13	1.5	1.3
Keyworker			
No	683	78.2	77.9
Health worker	31	3.6	3.5
Care worker	14	1.6	1.6
Other	145	16.6	17.1
IMD quintile			
1 (most deprived)	150	17.2	17.4
2	167	19.1	18.8
3	194	22.2	21.7
4	174	19.9	19.7
5 (least deprived)	188	21.5	22.5
Not answered			0.0
Household income			
Up to £19 999	178	20.4	19.8
£20 000–£34 999	206	23.6	23.6
£35 000–£59 999	172	19.7	19.6
Over £60 000	132	15.1	16.0
Not answered	185	21.2	21.1
Self-reported health status			
Very good	214	24.5	24.8
Good	416	47.7	47.9
Fair	176	20.2	19.8
Bad/very bad	60	6.9	6.7
Not answered	7	0.8	0.8
Health or disability issue in last 12 months			
Limited a lot	81	9.3	9.0
Limited a little	153	17.5	16.5
No	626	71.7	72.9
Not answered	13	1.5	1.6
Consider themselves vulnerable to COVID-19			
No	454	52.0	53.4
Yes	407	46.6	45.2
Not answered	12	1.4	1.5
Political party affiliation			
Conservative	250	28.6	28.1
Labour	311	35.6	36.0
Liberal democrat	67	7.7	7.5
Scottish National Party			0.0
UK Independence Party (UKIP)	24	2.8	2.7
Green	38	4.4	4.5
No affiliation	115	13.2	13.4
Other	14	1.6	1.6
Don't know	40	4.6	4.3
Not answered	14	1.6	1.9
Vote in 2016 EU referendum			
Remain	441	50.5	51.4
Leave	356	40.8	39.6
Didn't vote	65	7.5	7.8
Can’t remember	11	1.3	1.2

### Changes in trust in the Government to control the spread of COVID-19

**Figure 2:**
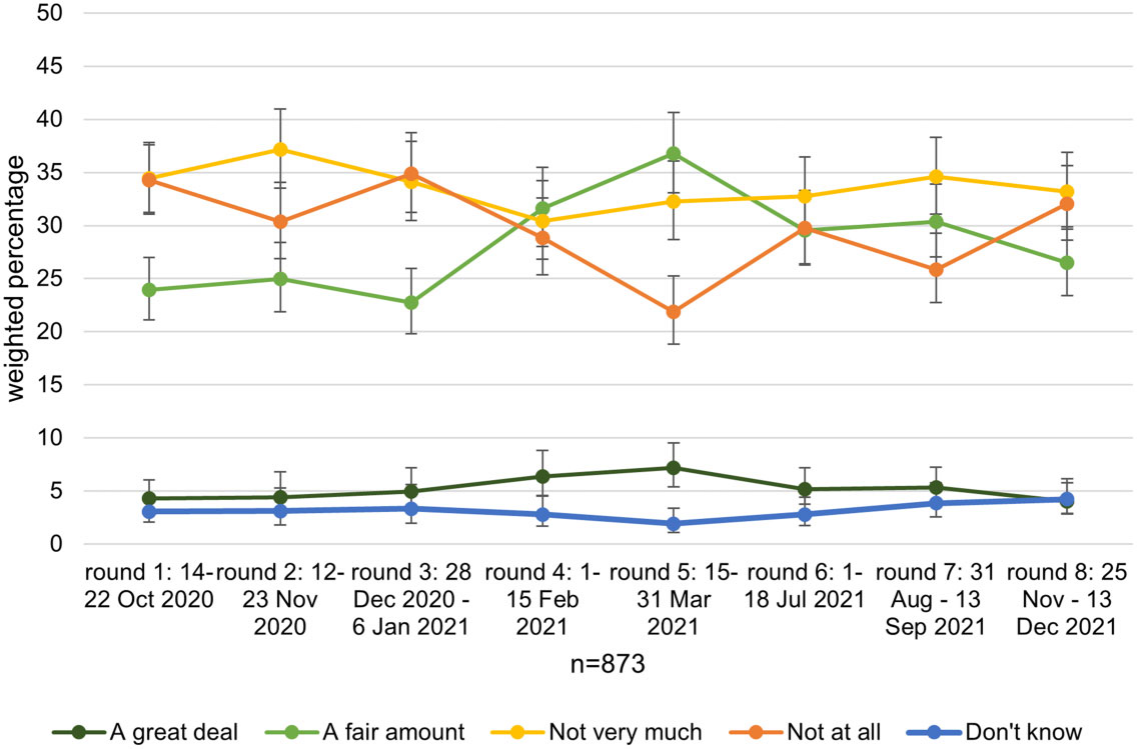
The extent of trust in the Government to control the spread of COVID-19, by survey round

Across all time points, most participants reported having no trust or not very much trust in the Government specifically to control the spread of COVID-19 (see figure 2). Trust in the Government was highest at survey round 5 (15 to 31 March 2021), when case numbers were lowest and restrictions started to be eased [44.0% (95% confidence interval [CI] 40.1–47.9) a great deal or fair amount vs. 54.1% (95% CI 50.2–58.0) not very much or not at all] and lowest at survey round 3 (28 December 2020 to 6 January 2021) when case numbers were highest just before the third national lockdown began (27.7% [95% CI 24.4–31.2] a great deal or fair amount vs. 70.0% [95% CI 65.3–72.5] not very much or not at all). The percentage of respondents reporting ‘don’t know’ ranged from 1.9% (95% CI 1.1–3.4) at survey round 5 to 4.2% (95% CI 2.9–6.1) at round 8.

### Factors associated with higher trust in the Government to control the spread of COVID-19

The results of the random-effects model are shown in table 2. Individuals were more likely to report a great deal or a fair amount of trust in the Government specifically to control COVID-19 at survey rounds 4, 5 and 7 compared with round 1. Older age groups [adjusted odds ratio (aOR) 3.75 (95% CI 1.82–7.71), *P* ≤ 0.001], participants with the lowest educational attainment [aOR 3.18 (95% CI 2.02–4.99), *P* ≤ 0.001], and those who reported that they generally supported the Conservative Party [aOR 9.17 (95% CI 5.6–15.12), *P* ≤ 0.001] were more likely to report having a great deal or a fair amount of trust. Conversely, individuals who were relatively less deprived [aOR 0.51 (95% CI 0.8–0.92), *P* = 0.02] and those who found the Government’s communications about what they should do in response to COVID-19 less clear were less likely to report a great deal or a fair amount of trust. Compared with those who thought that the measures taken by the Government to tackle COVID-19 were about right, both those that thought measures went too far [aOR 0.21 (95% CI 0.14–0.31), *P* ≤ 0.001] and those who thought they did not go far enough [aOR 0.12 (95% CI 0.09–0.16), *P* ≤ 0.001] were less likely to report trusting the Government to control COVID-19.

**Table 2:** Random-effects logistic regression results of public trust in English Government’s response to COVID-19 (*n* = 6413 observations)

	Observations across all survey rounds	Proportion with trust (a great deal or a fair amount)		aOR	95% CI	*P*-Value
		*n*	Weighted %			
Survey round						
1: 14–22 Oct 2020	813	239	29.43	1		
2: 12–23 Nov 2020	814	259	30.59	0.94	0.63–1.43	0.79
3: 28 Dec 2020 – 6 Jan 2021	813	262	29.16	0.89	0.59–1.35	0.59
4: 1–15 Feb 2021	818	345	40.01	1.88***	1.24–2.85	0.00
5: 15–31 Mar 2021	821	393	45.12	2.76***	1.80–4.21	0.00
6: 1–18 Jul 2021	815	315	35.73	1.37	0.90–2.10	0.14
7: 31 Aug – 13 Sep 2021	811	324	37.90	1.53**	1.00–2.34	0.05
8: 25 Nov – 13 Dec 2021	807	284	32.45	0.98	0.64–1.49	0.91
Age category						
18–29	838	186	22.78	1		
30–49	2315	703	30.91	1.72*	0.91–3.27	0.09
50–64	2113	891	40.68	2.81***	1.46–5.44	0.00
>65	1246	641	54.83	3.75***	1.82–7.71	0.00
Gender						
Male	3139	1261	39.99	0.96	0.65–1.41	0.82
Female	3373	1160	30.57	1		
IMD quintile						
1 (most deprived)	1128	471	38.04	1		
2	1222	501	39.52	1.18	0.65–2.14	0.59
3	1426	517	35.17	0.61	0.34–1.11	0.11
4	1300	460	32.58	0.54**	0.29–0.98	0.04
5 (least deprived)	1436	472	31.37	0.51**	0.28–0.92	0.02
Highest educational attainment						
Lower education	1727	936	53.34	3.18***	2.02–4.99	0.00
A-level or equivalent	990	353	34.02	1.47	0.86–2.53	0.16
Higher education	3795	1132	28.71	1		
Political affiliation						
Conservative	1924	1209	62.58	9.17***	5.56–15.12	0.00
Labour	2390	471	17.81	1		
Liberal Democrat	520	126	24.46	1.01	0.47–2.16	0.99
Green	291	47	20.95	0.87	0.31–2.44	0.80
No affiliation	818	301	37.83	2.50***	1.36–4.61	0.00
Other/don’t know	569	267	40.03	5.81***	2.92–11.58	0.00
Opinion on measures taken by Govt. to tackle COVID-19^a^						
Measures do not go far enough	3393	690	18.38	0.12***	0.09–0.16	0.00
Measures are about right	1845	1466	76.15	1		
Measures go too far	882	190	22.34	0.21***	0.14–0.31	0.00
Don’t know	392	75	17.65	0.17***	0.10–0.29	0.00
Clarity of Govt.‘s communications about what to do in response to COVID-19^a^						
Very clear	837	662	78.59	1		
Fairly clear	2528	1352	51.05	0.53***	0.36–0.77	0.00
Not very clear	1163	345	16.98	0.24***	0.16–0.37	0.00
Not at all clear	1163	38	3.33	0.12***	0.06–0.21	0.00
Don’t know	95	24	25.63	0.33**	0.13–0.85	0.02
Extent of trust in information provided by Govt. On COVID-19^a^						
A great deal	682	617	89.58	1		
A fair amount	2732	1564	54.85	0.12***	0.070–0.195	0.00
Not very much	1932	204	9.69	0.01***	0.006–0.019	0.00
Not at all	1072	20	1.94	0.00***	0.001–0.004	0.00
Don’t know	94	16	14.88	0.01**	0.004–0.030	0.00
Since completing last survey, had or currently have COVID-19^a^						
Had it, confirmed by a test	190	73	35.03	1		
Probably had it	309	121	40.40	0.78	0.34–1.82	0.57
Don’t know	767	252	30.95	0.59	0.28–1.23	0.16
Probably not had it	2056	646	30.34	0.64	0.32–1.28	0.21
Not had it	3091	1288	38.94	0.6	0.31–1.19	0.14
Constant term				16.27***	4.90–54.02	0.00
rho				0.55***	0.50–0.61	0.00
Log likelihood				−1758.68		
LR chi2(37)				1610.28***		
Observations	6413	2380	35			

a Time-varying; respondents were asked question each time surveyed.

* *P* ≤ 0.1,

** *P* ≤ 0.05,

*** *P* ≤ 0.001.

## Discussion

This study examined the public’s trust in the then Government to control the spread of COVID-19 in England after the first pandemic wave. Trust in the Conservative Government was found to be low throughout the period, with most respondents reporting they had no or not very much trust in the Government. Evidence from the first wave of the pandemic had shown a ‘rally-round-the-flag’ effect. However, by the time of the first survey round in autumn 2020, this had disappeared.^8^^,^^18^ A contributing factor was the revelation that in May 2020 Boris Johnson’s then chief-advisor, Dominic Cummings, had broken lockdown rules by travelling to Durham with his family who had COVID-19 symptoms.^19^ However, while the levels of trust in the Government specifically to control the spread of COVID-19 during the period of this study never reached the highs reported during the first wave of the pandemic, our results suggest that there may be scope for governments to avoid losing trust.^8^^,^^13^ Notable changes in trust were observed following survey rounds 3, 5 and 6.

The significant increase in trust observed after round 3 coincided with the rollout of the first vaccine and the introduction of the third national lockdown. The Government was widely perceived to have handled the rollout of the vaccine well and the UK’s vaccine programme was considered highly successful; it was the first country to start its mass vaccination programme and by round 4 of the survey, in February 2021, had administered more doses than any comparable country.^20^ Before the third national lockdown, introduced in January 2021, there had been rapidly rising infection rates and increasing divergence between Government actions and scientific advice.^21^ Opposition parties had also been calling for the Government to take further actions during autumn 2021 and widely condemned the decision on 19 December not to go ahead with the planned relaxation of rules for Christmas as having been made too late.^22^ The decision was reported in the mainstream media as a ‘major U-turn’ and accompanied by negative headlines that ‘Christmas had been cancelled’.^23^ These disparities between Government action and the scientific advice, and criticism from opposition parties, plus the negative portrayal of the Government’s handling in the media, likely contributed to the perception that the Government was not to be trusted to control the spread of COVID-19.^24^ It might also have served to damage opinion of the clarity of the Government’s communications on what to do in response to COVID-19, which we found to be a key determinant of trust. In contrast, the introduction of the third national lockdown, brought the Government’s actions more in line with public opinion; polling in January 2021 found that most of the public were supportive of the national lockdown (85%) and considered that it had come too late (77%).^25^ Our findings demonstrate that those who did not agree with the measures taken by the Government (either that they went too far or not far enough) had lower trust in the Government to control the spread of COVID-19.

Survey round 5 in spring 2021 showed the highest level of trust during our study period. It took place in the context of low case numbers and ahead of the Government’s four-step plan to lift the remaining restrictions.^10^ The decline in trust observed from survey round 6 potentially reflects dissatisfaction that the Government had pressed ahead with lifting all legal restrictions on 19 July despite rising case numbers as the Alpha and Delta variants took hold. By the last survey round, in winter 2021, the first allegations that parties took place at Downing Street and other Government buildings during the 2020 lockdowns (‘Partygate’) had emerged, which likely further corroded the public’s confidence and trust in the Government’s response, adding to the process of eroding trust which started with the Cummings affair in May 2020.^19^^,^^26^ Simultaneously, the Omicron variant started to spread and the Government reintroduced some restrictions as part of its Autumn and Winter Plan B. The mismatch between the Government’s earlier rhetoric that vaccines ‘will get us out of the pandemic’ and the rising case numbers, hospitalizations and deaths, potentially reduced trust as perceptions of performance did not meet people’s expectations.^27^

Participants who were younger, less deprived and more educated reported lower levels of trust in the Conservative Government to control the spread of COVID-19. The impact of age may have gained importance as a determinant of trust, given that the COVID-19 pandemic has been shown to have had a considerable impact on younger people and a negative impact on their confidence in political institutions and leaders.^28^^,^^29^

Pre-pandemic public trust in governmental institutions in general was found to increase with income-level and education.^30^ One potential reason why income and education were negatively associated with trust in the Government’s response in this study is political party affiliation. We found that those participants who affiliated themselves with the Conservative Party were more than nine times more likely to report higher levels of trust in the Government’s handling of the pandemic. Therefore, individuals who might more generally be expected to have had higher trust in Government, such as wealthier more highly educated individuals, may be influenced conversely when the Government is led by a party they do not support, in this case, Boris Johnson’s Conservative Government. The interesting finding in this context is that people who affiliated themselves with the Conservative Party—who tend to oppose state regulations and restrictions more than other parties—trusted the Conservative Government in handling the pandemic with lockdowns and other restricting measures. In the extraordinary context of the pandemic, partisan trust seemed to overcome libertarian views. Political partisanship is part of a wider trend in the UK, which has become more evident since the Brexit referendum.^31^^,^^32^ It has also been observed elsewhere, with approval ratings for governments’ responses to COVID-19 higher among those citizens who support the governing parties.^33^

### Strengths and limitations

This analysis took advantage of a longitudinal study conducted between October 2020 and December 2021, after the first pandemic wave in England. This design allowed us to examine changes in trust in the Government specifically to control the spread of COVID-19 over time and to capture the dynamic of the pandemic and the Government’s response to changing situations.

The survey was conducted online, using quota-sampling to recruit a representative sample of smartphone users from YouGov’s panel. Participants were younger and more highly educated than the general public and therefore less likely to vote conservative. This might have led to an overestimation of the level of distrust. Further, the study was conducted in English, thereby excluding a sub-group of the population with poor English skills. The question on the extent of trust in the Government to control the spread of COVID-19 could have been interpreted differently by the respondents, and responses may reflect conceptually distinct forms of trust such as general trust in politics, trust in national political leadership or COVID-19-specific trust, though the question was worded in an attempt to focus the response more narrowly on the public’s trust in the Government’s control of the pandemic. Thus, comparisons between studies asking questions about different aspects of trust in Government need to be drawn carefully.

### Implications

Trust in governments has previously been demonstrated to be an important prerequisite for the acceptance and adoption of protective measures in pandemics.^3–5^^,^^34^ Our findings that less than half of respondents trusted the Government to control the pandemic, potentially poses a significant barrier to the success of the UK’s response to the current and future pandemics, if it undermines the public’s adherence to Government recommendations. Evidence on the impact of trust on compliance during the current pandemic is complex because it appears to depend, at least in countries like the US and the UK, on people’s party political affiliations and how these relate to the Government of the day.^35^ Thus, where citizens are more concerned about action to control the spread of the virus than a laissez-faire Government and believe that the Government is not taking sufficient action, citizens might even increase their adherence beyond the recommended measures, irrespective of their level of trust in the Government’s response.^36^

In England during the period of the current study, it appears that to increase the public’s trust in the Government's response, there needed to be greater alignment between the Government’s actions, scientific advice and public opinion. The Johnson Government potentially overestimated the public’s appetite to ‘return to normal’. Given the higher levels of trust in scientists than the Government among the UK public,^37^ greater transparency in how evidence and advice are being used might also help foster trust in the Government’s ability to control the spread of COVID-19 and future pandemics.^38^ Tied to this is a need for clearer and more consistent communication of the Government’s response, to overcome what has been described as ‘alert fatigue’ whereby the public struggle to understand constantly changing rules.^39^

## Supplementary Material

ckad148_Supplementary_Data

## Data Availability

The data analyzed in this study are subject to licenses/restrictions and are available upon request. Requests to access these datasets should be directed to PIRU-pm@lshtm.ac.uk. Key points Throughout the study less than half of the study population in England reported that they trusted the Government specifically to control the spread of COVID-19; trust was lowest in winter 2020 when case numbers were highest just before the third national lockdown was introduced and highest in spring 2021 when case numbers were lowest, restrictions started to be eased and vaccinations to be rolled out. Periods of increasing trust were characterized by success (vaccination campaign), decreasing case numbers and hope for the lifting of restrictions, whereas periods of declining trust were characterized by contradiction (divergence from scientific advice, lifting restrictions despite increasing case numbers) and political scandals (i.e. that politicians and their advisers were not respecting the restrictions imposed on the rest of society). Participants who were younger, less deprived, more educated and who did not affiliate themselves with the Conservative Party reported lower levels of trust in the Conservative Government to control the spread of COVID-19. There may be scope to build (or not lose) trust where Government measures more closely align with public opinion, use of scientific advice is communicated transparently and measures are explained more clearly and consistently. However, it remains unclear, whether higher trust in the Government’s pandemic response would necessarily increase adherence to governmental measures in England because of the impact of political partisanship on this aspect of trust.

## References

[1] Prati G , Pietrantoni L, Zani B. Compliance with recommendations for pandemic influenza H1N1 2009: the role of trust and personal beliefs. Health Educ Res 2011; 26:761–9. 21613380 10.1093/her/cyr035

[2] Blair RA , Morse BS, Tsai LL. Public health and public trust: survey evidence from the Ebola Virus Disease epidemic in Liberia. Soc Sci Med 2017; 172:89–97. 27914936 10.1016/j.socscimed.2016.11.016

[3] Enria L , Waterlow N, Rogers NT, et al Trust and transparency in times of crisis: results from an online survey during the first wave (April 2020) of the COVID-19 epidemic in the UK. PLoS One 2021;16:e0239247. 33591985 10.1371/journal.pone.0239247 PMC7886216

[4] Weinberg J. Can political trust help to explain elite policy support and public behaviour in times of crisis? Evidence from the United Kingdom at the height of the 2020 coronavirus pandemic. Polit Stud 2022;70:655–79.

[5] Dohle S , Wingen T, Schreiber M. Acceptance and adoption of protective measures during the COVID-19 pandemic: the role of trust in politics and trust in Science. Soc Psychol Bull 2020;15:1–23.

[6] Chatagnier JT. The effect of trust in government on rallies ’round the flag. J Peace Res 2012;49:631–45.

[7] Bol D , Giani M, Blais A, Loewen PJ. The effect of COVID-19 lockdowns on political support: some good news for democracy? (Published in European Journal of Political Research) . SocArXiv 2020. Available at: https://osf.io/preprints/socarxiv/7hpj9/ (20 November 2022, date last accessed).

[8] Davies B , Lalot F, Peitz L, et al Changes in political trust in Britain during the COVID-19 pandemic in 2020: integrated public opinion evidence and implications. Humanit Soc Sci Commun 2021;8:1–9.

[9] Brown J , Kirk-Wade E, Baker C, Barber S. *Research Briefing: Coronavirus: A History of English Lockdown Laws*. Available at: https://researchbriefings.files.parliament.uk/documents/CBP-9068/CBP-9068.pdf (2 December 2022, date last accessed).

[10] Hale T , Anania J, Angrist N, et al *Variation in Government Responses to COVID-19. Version 12.0*. Blavatnik School of Government Working Paper. 2021. Available at: https://www.bsg.ox.ac.uk/research/publications/variation-government-responses-covid-19

[11] GOV.UK. *COVID-19 Response: Autumn and Winter Plan 2021*. Available at: https://www.gov.uk/government/publications/covid-19-response-autumn-and-winter-plan-2021 (8 January2023, date last accessed).

[12] Ipsos. *Coronavirus Data Trends – Britons’ Concern for the Country Continues to Decline*. Available at: https://www.ipsos.com/en-uk/coronavirus-data-trends-britons-concern-country-continues-decline (19 November 2022, date last accessed).

[13] Ipsos. *Survey on COVID-19 Track and Trace Smartphone App for the Health Foundation*. Available at: https://www.ipsos.com/en-uk/survey-covid-19-track-and-trace-smartphone-app-health-foundation (19 November 2022, date last accessed).

[14] Ofcom. Ofcom Nations & Regions Technology Tracker - 2019. technology-tracker-2019-uk-data-tables.pdf. Available at: https://www.ofcom.org.uk/__data/assets/pdf_file/0026/143981/technology-tracker-2019-uk-data-tables.pdf (8 August 2021, date last accessed).

[15] UK Government. *Education and Training Statistics for the UK, Reporting Year 2020 – Explore Education Statistics*. 2020. Available at: https://explore-education-statistics.service.gov.uk/find-statistics/education-and-training-statistics-for-the-uk/2020 (27 July 2021, date last accessed).

[16] ONS. *Population Estimates for the UK, England and Wales, Scotland and Northern Ireland*. 2020. Available at: https://www.ons.gov.uk/peoplepopulationandcommunity/populationandmigration/populationestimates/bulletins/annualmidyearpopulationestimates/latest#age-structure-of-the-uk-population (26 July 2021, date last accessed).

[17] Baker C , Uberoi E, Cracknell R. *General Election 2019: Full Results and Analysis*. Available at: https://commonslibrary.parliament.uk/research-briefings/cbp-8749/ (27 July 2021, date last accessed).

[18] Duffy B. the-handling-of-the-coronavirus-crisis.pdf. Available at: https://www.kcl.ac.uk/policy-institute/assets/the-handling-of-the-coronavirus-crisis.pdf (19 November 2022, date last accessed).

[19] Fancourt D , Steptoe A, Wright L. The Cummings effect: politics, trust, and behaviours during the COVID-19 pandemic. Lancet 2020;396:464–5. 32771083 10.1016/S0140-6736(20)31690-1 PMC7613216

[20] Baraniuk C. Covid-19: how the UK vaccine rollout delivered success, so far. BMJ 2021; 372:n421. 33602672 10.1136/bmj.n421

[21] SAGE. *Circuit Breakers: Implementing (Partial) Lockdown for 2 Weeks Over Half-Term*. 24 September 2020. GOV.UK. Available at: https://www.gov.uk/government/publications/circuit-breakers-implementing-partial-lockdown-for-2-weeks-over-half-term-24-september-2020 (1 August 2022, date last accessed).

[22] Starmer K. *Starmer Demands Urgent Review of Christmas COVID Rules*. The Labour Party. Available at: https://labour.org.uk/press/starmer-demands-urgent-review-of-christmas-covid-rules/ (19 November 2022, date last accessed).

[23] Murray W. “Christmas cancelled”: what the papers say as UK COVID bubbles burst. *The Guardian*. 2020. Available at: https://www.theguardian.com/world/2020/dec/20/christmas-cancelled-what-the-papers-say-as-covid-bubbles-burst (19 November 2022, date last accessed).

[24] National Audit Office (NAO). *Investigation into Government Procurement during the COVID-19 Pandemic - National Audit Office (NAO) Report*. Available at: https://www.nao.org.uk/reports/government-procurement-during-the-covid-19-pandemic/ (19 November 2022, date last accessed).

[25] YouGov. *Brits Support New National Lockdown* | YouGov. Available at: https://yougov.co.uk/topics/politics/articles-reports/2021/01/05/brits-support-national-lockdown-jan-2021 (19 November 2022, date last accessed).

[26] Ares M , Hernández E. The corrosive effect of corruption on trust in politicians: evidence from a natural experiment. Res Polit 2017;4:205316801771418.

[27] YouGov. *Government Approval*. Available at: https://yougov.co.uk/topics/politics/trackers/government-approval (10 August 2021, date last accessed).

[28] Aksoy C. *The Political Scar of Epidemics* | Systemic Risk Centre. Available at: https://www.systemicrisk.ac.uk/publications/discussion-papers/political-scar-epidemics (10 August 2021, date last accessed).

[29] Tasso AF , Hisli Sahin N, San Roman GJ. COVID-19 disruption on college students: academic and socioemotional implications. Psychol Trauma 2021;13:9–15. 33382329 10.1037/tra0000996

[30] OECD. *Statistical Insights: Trust in the United Kingdom - OECD*. Available at: https://www.oecd.org/sdd/statistical-insights-trust-in-the-united-kingdom.htm

[31] British Social Attitudes. *NatCen Social Research*. Available at: https://www.bsa.natcen.ac.uk/latest-report/british-social-attitudes-37/consequences-of-brexit.aspx (25 July 2022, date last accessed).

[32] Hobolt SB. The Brexit vote: a divided nation, a divided continent. J Eur Public Policy 2016;23:1259–77.

[33] Chen CWS , Fan TH. Public opinion concerning governments’ response to the COVID-19 pandemic. PLoS One 2022;17:e0260062. 35235561 10.1371/journal.pone.0260062 PMC8890629

[34] Han Q , Zheng B, Cristea M, et al Trust in government and its associations with health behaviour and prosocial behaviour during the COVID-19 pandemic. *PsyArXiv* 2020. Available at: https://psyarxiv.com/p5gns/

[35] Wiedemann J , Goldstein D. *Replication Data for: Who Do You Trust? The Consequences of Partisanship and Trust for Public Responsiveness to COVID-19 Orders*. Harvard Dataverse. 2021. Available at: https://dataverse.harvard.edu/dataset.xhtml?persistentId=doi:10.7910/DVN/CEJAHP (19 November 2022, date last accessed).

[36] Guglielmi S , Dotti Sani GM, Molteni F, et al Public acceptability of containment measures during the COVID-19 pandemic in Italy: how institutional confidence and specific political support matter. IJSSP 2020;40:1069–85.

[37] Duffy B. *Coronavirus Fallout: Blame, Trust and the Future of the UK*. Available at: https://www.kcl.ac.uk/news/coronavirus-fallout-blame-trust-and-the-future-of-the-uk (19 November 2022, date last accessed).

[38] Haddon C , Sasse T, Nice A. *Science Advice in a Crisis*. 18 December 2020. Available at: https://www.instituteforgovernment.org.uk/publications/science-advice-crisis (3 December 2022, date last accessed).

[39] Williams SN , Armitage CJ, Tampe T, Dienes KA. Public perceptions of non-adherence to pandemic protection measures by self and others: a study of COVID-19 in the United Kingdom. PLoS One 2021;16:e0258781. 34710125 10.1371/journal.pone.0258781 PMC8553167

