# Supplementary material for: Public trust in the Government to control the spread of COVID-19 in England after the first wave—a longitudinal analysis
Source: Eur J Public Health. 2023 Aug 14;33(6):1155–62. doi: 10.1093/eurpub/ckad148 (PMC10710334; doi:10.1093/eurpub/ckad148)
Supplement: ckad148_Supplementary_Data [file ckad148_supplementary_data.pdf]

Supplementary files

Model specification

The random-effect model estimated is empirically stated as (40):

$$Trust_{it} = \propto + \beta X_{it} + \mu_i + \varepsilon_{it} \qquad (1)$$

Where:

i represent individual respondents, i= 1, 2, ………N;

t represent survey round (time), t=1, 2, …….T;

β is a vector of parameters to be estimated;

X_{it} is a vector of explanatory variables included in the model;

ε_{it} is the error term for both cross section and time series; and

μ_i is the individual residual, that is the random characteristics of the *i*th respondent that remains at all times.

Statistical analysis

Table S- 1 Variables examined

Variables	Definition and measurement
Dependent variable	
Trust in government	Level of trust in governments to control the spread of COVID-19 during the pandemic.<br>A dummy variable, measures as 1 (more trusting) if a respondent selected "a great deal of trust" or "a fair amount of trust"; and 0 (less trusting) if survey respondents selected "not very much trust" or "not at all trust".
Explanatory variables	
Survey rounds	The survey period in which data were collected from respondents.<br>Dummy variables:<br>W1 - 1 if the data collection was done from 14th – 22nd October 2020; and 0, otherwise;<br>W2 - 1 if the data collection was done from 12th- 23rd November 2020; and 0, otherwise;<br>W3 - 1 if the data collection was done from 28th December 2020 – 6th January 2021; and 0, otherwise;<br>W4 - 1 if the data collection was done from 1st – 15th February 2021; and 0 otherwise.<br>W5 - 1 if the data collection was done from 15th – 31st March 2021; and 0, otherwise;<br>W6 - 1 if the data collection was done from 1st – 18th July 2021; and 0, otherwise;<br>W7 - 1 if the data collection was done from 31st August – 13th September 2021; and 0, otherwise;<br>W8 - 1 if the data collection was done from 25th November – 13th December 2021; and 0, otherwise
Age	Age of respondents in years. This was categorized into four age groups, and each age group was measured as a dummy variable as follows:<br>18-29 - 1 if the respondent's age belongs to this age group, and 0, otherwise;<br>30-49 - 1 if the respondent's age belongs to this age group, and 0, otherwise;

Variables	Definition and measurement
	50-64 - 1 if the respondent's age belongs to this age group, and 0, otherwise;<br>>=65 - 1 if the respondent's age belongs to this age group, and 0, otherwise
Gender	Sex of respondent. A dummy variable, measure as 1 if the respondent was a male and 0, otherwise.
IMD	Level of multiple deprivation of respondents based on their geographical location and local authority district to which they belong. This was categorized into 5 groups, with 1 being the most deprived and 5 being the least derived. Each of the categories was measured as a dummy variable:<br>1-most deprived – 1 if the respondent belong to this category and 0, otherwise;<br>2 - 1 if the respondent belong to this category and 0, otherwise;<br>3 - 1 if the respondent belong to this category and 0, otherwise;<br>4 - 1 if the respondent belong to this category and 0, otherwise;<br>5- least deprived - 1 if the respondent belong to this category and 0, otherwise;
Educational level	Highest educational qualification of respondents. This was categorized into three, and each category was measured as a dummy variable.<br>Lower – 1 if the respondent had no formal qualifications, GCSE or equivalent, other educational qualifications, and 0, otherwise;<br>A-level or equivalent -1 if the respondent had A-levels of equivalent qualifications, and 0, otherwise;<br>Higher education -1 if the respondent had a university educational qualification, and 0, otherwise;
Political affiliation	Political affiliation of respondents. A dummy variable, measured as:<br>Conservatives – 1 if the respondent is affiliated to Conservatives party and 0, otherwise<br>Labour - 1 if the respondent is affiliated to Labour party and 0, otherwise<br>Liberal Democrats - 1 if the respondent is affiliated to Liberal Democrats party and 0, otherwise<br>Green - 1 if the respondent is affiliated to Green party and 0, otherwise<br>No affiliation - 1 if the respondent is not affiliated to any political party and 0, otherwise<br>Other/don't know - 1 if the respondent belongs to any other political party and 0, otherwise
Opinion on measures	Respondents' view on measure put in place by English government to control the spread of COVID-19 pandemic. A dummy variable, measured as:<br>Measures do not go far enough – 1 if the respondent think the COVID-19 measures do not go far enough, and 0, otherwise<br>Measures are about right - 1 if the respondent think the COVID-19 measures are about right, and 0, otherwise<br>Measures go too far - 1 if the respondent think the COVID-19 measures go too far, and 0, otherwise<br>Don't know -1 if the respondent has no opinion on government COVID-19 measures, and 0, otherwise
Clarity of communication	Respondents' opinion about how clear they found English government's communications about what they should do in response to COVID-19. A dummy variable, measured as:<br>Very clear – 1 if the respondent found government communications very clear, and 0, otherwise;<br>Fairly clear - 1 if the respondent found government communications fairly clear, and 0, otherwise;<br>Not very clear - 1 if the respondent found government communications not very clear, and 0, otherwise;<br>Not at all clear - 1 if the respondent found government communications not at all clear, and 0, otherwise;<br>Don't know - 1 if the respondent don't know if government communication was clear or not, and 0, otherwise;

Variables	Definition and measurement
Trust in information provided	Trust in information provided by the English government on COVID-19. A dummy variable, measured as:<br>A great deal – 1 if the respondent has a great deal of trust in information provided by government on COVID-19, and 0, otherwise;<br>A fair amount - 1 if the respondent has a fair amount of trust in information provided by government on COVID-19, and 0, otherwise;<br>Not very much - 1 if the respondent does not very much trust information provided by government on COVID-19, and 0, otherwise;<br>Not at all - 1 if the respondent does not have trust at all in information provided by government on COVID-19, and 0, otherwise;<br>Don't know – 1 if the respondent is indifferent about trust in information provided by government on COVID-19, and 0, otherwise.
COVID-19 infection	Whether the respondent think they have had before, or having COVID-19 during the interview. A dummy variable, measured as:<br>I've definitely had it and had it confirmed by a test – 1 if the respondent has had COVID-19 which was confirmed by a test, and 0, otherwise;<br>I think I've probably had it – 1 if the respondent thinks he/she has probably ha COVID-19, and 0, otherwise;<br>I don't know whether I've had it or not – 1 if the respondent doesn't know whether he/she has had COVID-19 or not, and 0, otherwise;<br>I think I've probably not had it – 1 if the respondent thinks he/she has probably not had COVID-19, and 0, otherwise;<br>I've definitely not had it – 1 if the respondent thinks he/she has definitely not had COVID-19, and 0,otherwise.

Additional results

Response rate

Table S- 2 Response rate at each round of the survey and the total number of surveys participants responded to

		Survey round(s)							
		1	2	3	4	5	6	7	8
Total number of respondents at each survey round	n	1,899	1,672	1,619	1,568	1,508	1,368	1,229	1,149
	%	100%	88%	85%	83%	79%	72%	65%	61%
Total number of survey rounds responded to	n	74	90	84	106	215	180	277	873
	%	4%	5%	4%	5%	11%	9%	15%	46%

Univariate analysis

Table S- 3 bivariate logistic regression examining association between trust in government to control the spread of COVID-19 and explanatory variables, for the balanced sample (n=6,704 observations)

	Observations across all rounds	Proportion with trust (a great deal or a fair amount)		p-Value (Pseudo R2)
		n	%	
Age category				
18-29	880	186	21.14%	0.00 (0.03)
30-49	2,392	703	29.39%	
50-64	2,168	891	41.10%	
>65	1,264	641	50.71%	
Gender				
Female	3,512	1,160	33.03%	0.00 (0.003)
Male	3,192	1,261	39.51%	
IMD quintile				
1 - most deprived	1,150	471	40.96%	0.00 (0.004)
2	1,272	501	39.39%	
3	1,467	517	35.24%	
4	1,355	460	33.95%	
5 - least deprived	1,460	472	32.33%	
Highest educational attainment				
Lower education	1,784	936	52.47%	0.00 (0.04)
A-level or equivalent	1,024	353	34.47%	
Higher education	3,896	1,132	29.06%	
Political affiliation				
Conservative	1,960	1,209	61.68%	0.00 (0.12)
Labour	2,448	471	19.24%	
Liberal Democrat	528	126	23.86%	
Green	304	47	15.46%	

	Observations across all rounds	Proportion with trust (a great deal or a fair amount)		p-Value (Pseudo R2)
		n	%	
No affiliation	872	301	34.52%	
Other/don't know	592	267	45.10%	
Opinion on measures taken by Govt. to tackle COVID-19[i]				
Measures do not go far enough	3,432	690	20.10%	0.00 (0.23)
Measures are about right	1,878	1,466	78.06%	
Measures go too far	917	190	20.72%	
Don't know	477	75	15.72%	
Clarity of Govt.'s communications about what to do in response to COVID-19[i]				
Very clear	850	662	77.88%	0.00 (0.23)
Fairly clear	2,604	1,352	51.92%	
Not very clear	1,925	345	17.92%	
Not at all clear	1,173	38	3.24%	
Don't know	152	24	15.79%	
Extent trust information provided by Govt. On COVID-19[i]				
A great deal	687	617	89.81%	0.00 (0.33)
A fair amount	2,777	1,564	56.32%	
Not very much	1,946	204	10.48%	
Not at all	1,084	20	1.85%	
Don't know	210	16	7.62%	
ONS area classification				
Urban	5,336	1,906	35.72%	0.29
Town and Fringe	608	235	38.65%	
Rural	752	273	36.30%	
Ethnicity				
White	5,984	2,144	35.83%	0.08
All other ethnic groups	720	277	38.47%	

	Observations across all rounds	Proportion with trust (a great deal or a fair amount)		p-Value (Pseudo R2)
		n	%	
Self-reported health status				
Very good/good	4,712	1,739	36.91%	0.01 (0.001)
Fair	1,426	472	33.10%	
Bad/very bad	496	197	39.72%	
Not answered	70	13	18.57%	
Any underlying condition making them vulnerable to COVID-19				
No	3,512	1,147	32.66%	0.00 (0.004)
Yes	3,112	1,247	40.07%	
Keyworker				
Non-keyworker	5,280	1,968	37.27%	0.0004 (0.001)
Keyworker	1,424	453	31.81%	
Concern risk of COVID-19 poses to self				
Very concerned	1,230	460	37.40%	0.00 (0.003)
Fairly concerned	3,074	1,162	37.80%	
Not very concerned	1,832	644	35.15%	
Not at all concerned	522	142	27.20%	
Don’t know	46	13	28.26%	
Concern risk of COVID-19 poses to country				
Very concerned	2,447	766	31.30%	0.00 (0.01)
Fairly concerned	3,290	1,328	40.36%	
Not very concerned	706	267	37.82%	
Not at all concerned	228	52	22.81%	
Don’t know	33	8	24.24%	
Employment Status				
Currently working	3,181	1,088	34.20%	0.00 (0.01)
Not working/unemployed/permanently sick	817	288	35.25%	

	Observations across all rounds	Proportion with trust (a great deal or a fair amount)		p-Value (Pseudo R2)
		n	%	
Voluntary/home work/other	395	120	30.38%	
Education	101	12	11.88%	
Retired	1,406	660	46.94%	
Household Income				
Up to £19,999	1,360	584	42.94%	0.00 (0.01)
£20,000 - £34,999	1,600	558	34.88%	
£35,000-£59,999	1,336	511	38.25%	
£60,000 and over	1,048	312	29.77%	
Vote in 2016 EU referendum				
Remain	3,448	796	23.09%	0.00 (0.07)
Leave	2,728	1,428	52.35%	
Did not vote/can't remember	528	197	37.31%	
Since completing last survey, had or currently have COVID-19[i]				
Definitely had it and confirmed by a test	190	73	35.42%	0.00 (0.01)
Probably had it	309	121	29.16%	
Don’t know	767	252	32.86%	
Probably not had it	2,056	646	31.42%	
Definitely not had it	3,091	1,288	41.67%	

Note: OR=Odds Ratio, CI= confidence interval

i time-varying; respondents were asked question each time surveyed

Descriptive analysis

Figure S- 1 The extent trust government to control the spread of COVID-19, by survey round

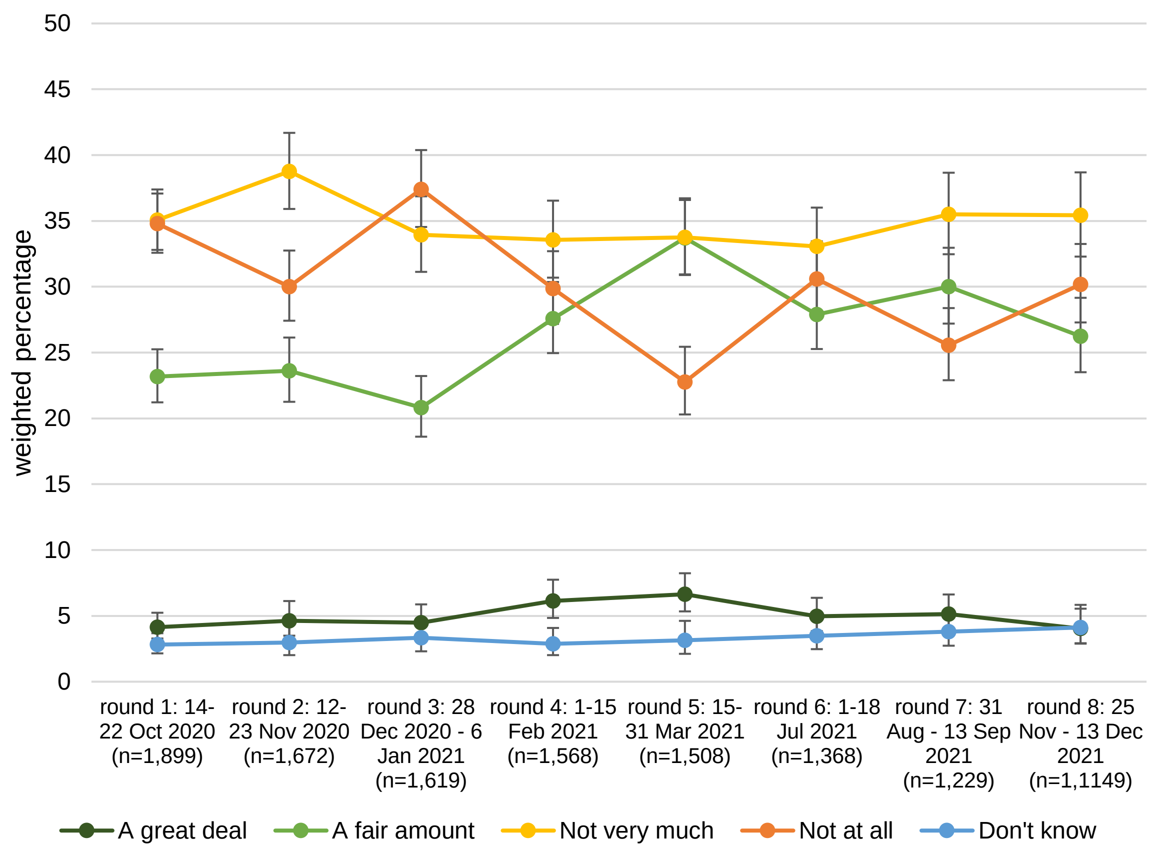

Multivariate regression

Table S- 4 Random effects logistic regression results of public trust in English Government's response to COVID-19 (n=10,785)

	Observations across all rounds	Proportion with trust (a great deal or a fair amount)		aOR	95%CI	p-value
		n	Weighted %			
Survey round						
1: 14-22 Oct 2020	1,695	493	28.88%	1		
2: 12-23 Nov 2020	1,511	476	29.83%	0.86	0.64 -1.16	0.33
3: 28 Dec 2020 – 6 Jan 2021	1,486	454	27.07%	0.76*	0.56 -1.02	0.07
4: 1-15 Feb 2021	1,445	564	35.83%	1.28	0.95 -1.72	0.10
5: 15-31 Mar 2021	1,393	636	42.44%	1.86***	1.37 -2.52	0.00
6: 1-18 Jul 2021	1,260	461	34.51%	0.95	0.70 -1.30	0.76
7: 31 Aug – 13 Sep 2021	1,131	447	37.35%	1.21	0.88 -1.67	0.24
8: 25 Nov – 13 Dec 2021	1,065	367	32.23%	0.73*	0.52 -1.01	0.06
Age category						
18-29	1,915	419	22.44%	1		
30-49	4,075	1,271	31.55%	1.90***	1.29 - 2.78	0.00
50-64	3,163	1,305	40.13%	2.62***	1.73 - 3.98	0.00
>65	1,833	903	50.49%	3.20***	2.00 -5.14	0.00
Gender						
Male	5,074	1,910	36.82%	0.82	0.62 -1.07	0.14
Female	5,912	1,988	30.27%	1		
IMD quintile						
1 - most deprived	1,887	684	34.31%	1		
2	2,011	737	34.91%	0.99	0.64 -1.51	0.95
3	2,300	815	33.02%	0.79	0.52 -1.20	0.27
4	2,353	856	34.27%	0.77	0.50 -1.16	0.21
5 - least deprived	2,435	806	30.40%	0.72	0.47 -1.08	0.12
Highest educational attainment						
Lower education	2,617	1,323	48.90%	2.28***	1.65 -3.15	0.00
A-level or equivalent	1,607	560	31.71%	1.25	0.85 -1.82	0.25
Higher education	6,762	2,015	28.93%	1		
Political affiliation						
Conservative	3,110	1,930	62.15%	7.36***	5.16 -10.5	0.00
Labour	3,919	768	18.04%	1		
Liberal Democrat	1,031	245	23.86%	0.89	0.54 -1.45	0.63
Green	608	98	17.70%	0.8	0.42 -1.55	0.52
No affiliation	1,301	468	35.16%	2.14***	1.38 -3.31	0.00
Other/don't know	1,017	389	33.05%	3.43***	2.15 -5.47	0.00
Opinion on measures taken by Govt. to tackle the COVID-19 outbreak[i]						
Measures do not go far enough	5,884	1,064	16.30%	0.11***	0.09 -0.13	0.00
Measures are about right	3,030	2,371	74.23%	1		
Measures go too far	1,405	339	25.30%	0.23***	0.17 -0.31	0.00

	Observations across all rounds	Proportion with trust (a great deal or a fair amount)		aOR	95%CI	p-value
		n	Weighted %			
Don't know	667	124	18.16%	0.18***	0.13 -0.27	0.00
Clarity of Govt.'s communications about what to do in response to COVID-19[i]						
Very clear	1,468	1,174	78.25%	1		
Fairly clear	4,029	2,094	48.92%	0.48***	0.37 -0.64	0.00
Not very clear	3,318	544	15.45%	0.19***	0.14 -0.26	0.00
Not at all clear	2,022	56	2.60%	0.07***	0.05 -0.11	0.00
Don't know	149	30	20.52%	0.21***	0.10 -0.45	0.00
Extent trust information provided by Govt. On COVID-19[i]						
A great deal	1,218	1,072	84.91%	1		
A fair amount	4,576	2,438	49.88%	0.17***	0.12 -0.23	0.00
Not very much	3,244	332	9.53%	0.02***	0.01 -0.02	0.00
Not at all	1,789	27	1.64%	0.00***	0.001 -0.004	0.00
Don't know	159	29	14.74%	0.03***	0.01 -0.05	0.00
Since completing last survey, had or currently have COVID-19[i]						
Definitely had it and confirmed by a test	327	113	32.11%	1		
Probably had it	623	215	33.67%	0.95	0.51 -1.77	0.88
Don't know	1,440	454	29.52%	0.76	0.44 -1.32	0.33
Probably not had it	3,609	1,110	29.05%	0.78	0.47 -1.31	0.35
Definitely not had it	4,786	1,938	38.27%	0.77	0.46 -1.27	0.30
Constant term				17.16***	7.57 -38.88	0.00
rho				0.52***	0.48 -0.56	0.00
Log likelihood				-3042.83		
Wald chi2(37)				1548.06***		
Observations	10,785	3898	33.26%			

Note: aOR=adjusted Odds Ratio, CI= confidence interval

i time-varying; respondents were asked question each time surveyed

* p≤0·1, ** p≤0·05, *** p≤0·001

Exploration of data with random effect panel ordered logistic regression model

Dependent variable: Trust [1=not at all; 2=not very much; 3=a fair amount; and 4=A great deal]

Explanatory variables are the same as those defined above (Table S-1).

Multivariate regression – Balanced data

Table S-5 Random effects ordered logistic regression results of public trust in English Government's response to COVID-19 (n=6,413)

Variable	aOR	95% CI	z-Statistics	P-Value
Survey round				
1: 14-22 Oct 2020	1			
2: 12-23 Nov 2020	0.88	0.68-1.14	-0.96	0.34
3: 28 Dec 2020 – 6 Jan 2021	0.77*	0.59-1.00	-1.95	0.05
4: 1-15 Feb 2021	1.15	0.88-1.50	1.03	0.30
5: 15-31 Mar 2021	1.97***	1.50-2.57	4.91	0.00
6: 1-18 Jul 2021	1.00	0.77-1.30	0.00	1.00
7: 31 Aug – 13 Sep 2021	1.38**	1.06-1.80	2.38	0.02
8: 25 Nov – 13 Dec 2021	0.87	0.67-1.14	-1.02	0.31
Age category				
18-29	1			
30-49	1.21	0.73-2.03	0.74	0.46
50-64	1.74**	1.02-2.97	2.05	0.04
>65	2.68***	1.49-4.82	3.29	0.00
Gender				
Male	0.94	0.68-1.30	-0.38	0.70
Female	1			
IMD quintile				
1 - most deprived	1			
2	1.10	0.69-1.76	0.40	0.69
3	0.63*	0.39-1.01	-1.92	0.06
4	0.59**	0.37-0.94	-2.19	0.03
5 - least deprived	0.54**	0.34-0.88	-2.51	0.01
Highest educational attainment				
Lower education	2.94***	2.01-4.32	5.52	0.00
A-level or equivalent	1.40	0.89-2.20	1.46	0.15
Higher education	1			
Political affiliation				
Conservative	9.59***	6.32-14.54	10.64	0.00
Labour	1			
Liberal Democrat	1.66	0.90-3.04	1.63	0.10
Green	0.80	0.36-1.78	-0.54	0.59
No affiliation	2.67***	1.61-4.45	3.78	0.00
Other/don't know	5.20***	2.88-9.39	5.46	0.00
Opinion on measures taken by Govt. to tackle the COVID-19 outbreak[i]				
Measures do not go far enough	0.18***	0.15-0.23	-15.28	0.00

Variable	aOR	95% CI	z-Statistics	P-Value
Measures are about right	1			
Measures go too far	0.30***	0.22-0.41	-7.74	0.00
Don't know	0.40***	0.28-0.56	-5.30	0.00
Clarity of Govt.'s communications about what to do in response to COVID-19[i]				
Very clear	1			
Fairly clear	0.58***	0.45-0.76	-4.04	0.00
Not very clear	0.28***	0.21-0.38	-8.22	0.00
Not at all clear	0.12***	0.09-0.18	-11.05	0.00
Don't know	0.35***	0.18-0.69	-3.08	0.00
Extent trust information provided by Govt. On COVID-19[i]				
A great deal	1			
A fair amount	0.04***	0.03-0.06	-18.39	0.00
Not very much	0.01***	0.00-0.01	-24.33	0.00
Not at all	0.00***	0.00-0.00	-29.84	0.00
Don't know	0.01***	0.01-0.02	-13.32	0.00
Since completing last survey, had or currently have COVID-19[i]				
Definitely had it and confirmed by a test	1			
Probably had it	1.32	0.75-2.34	0.97	0.33
Don't know	1.18	0.72-1.93	0.65	0.52
Probably not had it	1.25	0.79-1.98	0.94	0.35
Definitely not had it	1.16	0.73-1.82	0.62	0.53
/cut1	-7.43	-8.32--6.54		
/cut2	-2.91	-3.79--2.04		
/cut3	3.42	2.55-4.29		
/sigma2_u	4.15	3.56-4.85		
Log likelihood	-4074.9081			
LR chi2(37)	2390.57***			
Observation	6,413			

Note: aOR=adjusted Odds Ratio, CI= confidence interval

i time-varying; respondents were asked question each time surveyed

* p≤0·1, ** p≤0·05, *** p≤0·001

Multivariate regression - Unbalanced data

Table S-6 Random effects ordered logistic regression results of public trust in English Government's response to COVID-19 (n=10,785)

Variable	aOR	[95% CI	z-statistic	P-value
Survey round				
1: 14-22 Oct 2020	1			
2: 12-23 Nov 2020	0.97	0.81-1.17	-0.3	0.76
3: 28 Dec 2020 – 6 Jan 2021	0.68***	0.57-0.82	-4.0	0.00
4: 1-15 Feb 2021	1.05	0.87-1.27	0.5	0.60
5: 15-31 Mar 2021	1.72***	1.41-2.09	5.4	0.00
6: 1-18 Jul 2021	0.85	0.70-1.04	-1.6	0.11
7: 31 Aug – 13 Sep 2021	1.16	0.95-1.42	1.4	0.15
8: 25 Nov – 13 Dec 2021	0.77**	0.63-0.95	-2.4	0.02
Age category				
18-29	1			
30-49	1.62***	1.18-2.21	3.0	0.00
50-64	2.07***	1.47-2.92	4.2	0.00
>65	2.84***	1.92-4.22	5.2	0.00
Gender				
Male	0.82	0.65-1.02	-1.8	0.08
Female	1			
IMD quintile				
1 - most deprived	1			
2	0.89	0.64-1.25	-0.7	0.51
3	0.75	0.53-1.05	-1.7	0.09
4	0.74	0.53-1.03	-1.8	0.07
5 - least deprived	0.66	0.47-0.92	-2.4	0.02
Highest educational attainment				
Lower education	2.45***	1.86-3.24	6.3	0.00
A-level or equivalent	1.43	1.04-1.96	2.2	0.03
Higher education	1			
Political affiliation				
Conservative	8.25***	6.10-11.15	13.7	0.00
Labour	1			
Liberal Democrat	1.26	0.85-1.88	1.1	0.26
Green	0.78	0.47-1.29	-1.0	0.34
No affiliation	2.28***	1.57-3.31	4.4	0.00
Other/don't know	2.99***	2.00-4.46	5.4	0.00
Opinion on measures taken by Govt. to tackle COVID-19[i]				
Measures do not go far enough	0.17***	0.14-0.20	-21.3	0.00
Measures are about right	1			
Measures go too far	0.33***	0.26-0.41	-9.5	0.00
Don't know	0.38***	0.30-0.50	-7.3	0.00
Clarity of Govt.'s communications about what to do in response to COVID-19[i]				
Very clear	1			
Fairly clear	0.48***	0.40-0.59	-7.2	0.00
Not very clear	0.23***	0.18-0.29	-12.7	0.00

Variable	aOR	[95% CI	z-statistic	P-value
Not at all clear	0.10***	0.07-0.13	-16.4	0.00
Don't know	0.27***	0.16-0.46	-4.9	0.00
Extent of trust in information provided by Govt. On COVID-19[i]				
A great deal	1			
A fair amount	0.06***	0.05-0.08	-22.7	0.00
Not very much	0.01***	0.01-0.01	-30.9	0.00
Not at all	0.00***	0.00-0.00	-39.2	0.00
Don't know	0.02***	0.01-0.04	-15.0	0.00
Since completing last survey, had or currently have COVID-19[i]				
Had it, confirmed by a test	1			
Probably had it	1.17	0.77-1.78	0.7	0.47
Don't know	1.01	0.69-1.46	0.0	0.97
Probably not had it	1.10	0.78-1.56	0.6	0.58
Not had it	1.08	0.77-1.52	0.4	0.66
/cut1	-7.40***	-8.01--6.78		
/cut2	-2.91***	-3.52--2.31		
/cut3	3.00***	2.40-3.60		
/sigma2_u	3.97***	3.52-4.47		
Log likelihood	-7115.49			
Wald chi(37)	3232.75***			
Observation	10,785			

Note: aOR=adjusted Odds Ratio, CI= confidence interval

i time-varying; respondents were asked question each time surveyed

* p≤0·1, ** p≤0·05, *** p≤0·001
